# Improved prostate diffusion imaging using deep learning denoising and phase correction with ultra-high-density coil array

**DOI:** 10.1093/radadv/umag019

**Published:** 2026-03-28

**Authors:** Sherry S Huang, Xinzeng Wang, Patricia Lan, Milica Medved, Nurullah Kaya, Clyve K Follante, Yunjeong Stickle, Jonathan Taylor, Ambereen Yousuf, Roger Engelmann, Fraser J L Robb, Arnaud Guidon, Grace Lee, Aytekin Oto

**Affiliations:** Research and Scientific Affairs, GE HealthCare, Rochester, MN, 55901, United States; MR Clinical Solutions & Research Collaborations, GE HealthCare, Houston, TX, 77030, United States; MR Clinical Solutions & Research Collaborations, GE HealthCare, Menlo Park, CA, 94025, United States; Department of Radiology, University of Chicago, Chicago, IL, 60637, United States; Department of Radiology, University of Chicago, Chicago, IL, 60637, United States; MR Coils, GE HealthCare, Aurora, OH, 44202, United States; MR Coils, GE HealthCare, Aurora, OH, 44202, United States; Department of Radiology, University of Chicago, Chicago, IL, 60637, United States; Department of Radiology, University of Chicago, Chicago, IL, 60637, United States; Department of Radiology, University of Chicago, Chicago, IL, 60637, United States; MR Clinical Solutions & Research Collaborations, GE HealthCare, Aurora, OH, 44202, United States; MR Clinical Solutions & Research Collaborations, GE HealthCare, Boston, MA, 02215, United States; Department of Radiology, University of Chicago, Chicago, IL, 60637, United States; Department of Radiology, University of Chicago, Chicago, IL, 60637, United States

**Keywords:** prostate MRI, diffusion weighted imaging, deep-learning reconstruction, deep-learning denoising, phase correction, flexible-coil-array

## Abstract

**Background:**

MR diffusion-weighted imaging (DWI), especially at high *b*-value, is a key acquisition to help identify clinically significant prostate cancer; however, it suffers from low signal-to-noise ratio (SNR), high noise floor, and susceptibility artifact.

**Purpose:**

To demonstrate the feasibility of improving DWI quality using a novel 50-channel pelvic coil in conjunction with a deep learning (DL)-based phase correction and a DL-denoising algorithm.

**Methods:**

In this prospective, single-center study, 24 consecutive men referred for prostate multiparametric MRI over 16 months were enrolled (age 47–79 years; mean, 68.1 years). Axial T2-weighted images and DWI were obtained using a prototype 50‑channel coil and standard clinical phased array (3 T Architect, GE HealthCare, USA). The DWI acquisitions were reconstructed with the vendor’s deep learning denoising algorithm (ARDL). The same raw data were reconstructed offline using an investigational DL Phase Correction algorithm with ARDL (DLPC+ARDL). Two independent readers scored DWI and ADC series using 4 qualitative criteria. SNR and contrast-to-noise ratio (CNR) were measured on *b* = 1500 s/mm^2^ images. Combined reader scores were compared using the Wilcoxon matched‑pairs signed‑rank test, inter‑reader variability was assessed using Cohen’s κ, and quantitative SNR/CNR values were compared using 2‑tailed paired t‑tests.

**Results:**

Twenty men were analyzable for qualitative and 18 for quantitative metrics (reported as mean ± SD). 50‑channel pelvic coil with DLPC+ARDL produced the highest SNR (99.70 ± 28.50) and CNR (91.68 ± 44.39), exceeding 50‑channel with ARDL alone (SNR 56.44 ± 28.50; CNR 51.11 ± 28.67), 30‑channel anterior array with ARDL (SNR 31.41 ± 13.18; CNR 26.26 ± 13.52), and DLPC + ARDL (SNR 49.4 ± 18.6; CNR 44.7 ± 18.3) (all *P* < .0001). Reader scores favored DLPC+ARDL in prostate border definition, peripheral/transition zone distinction, lesion conspicuity, and confidence of extraprostatic extension (all *P* values for DWI at *b* = 1500 s/mm^2^ < 0.0001; synthetic DWI at *b* = 2000 s/mm^2^: *P *= 7.5 × 10^−7^–0.01). Inter‑reader agreement was fair for acquired DWI (quadratic‑weighted κ = 0.34) and lower for synthetic DWI (κ = 0.19).

**Conclusion:**

DWI images acquired using the 50‑channel pelvic coil and reconstructed with the DLPC + ARDL pipeline yield the highest image quality compared to ARDL only pipeline and to all 30-channel coil imaging.

**Abbreviations** 

AA, anterior array; ADC, apparent diffusion coefficient; ARDL, AIR Recon deep learning; CNR, contrast-to-noise ratio; DL, deep learning; DLPC, deep learning–based phase correction; DWI, diffusion‑weighted imaging; EPE, extraprostatic extension; mpMRI, multiparametric MRI; SNR, signal-to-noise ratio

**Summary** 

This proof-of-concept prospective feasibility study demonstrates that combining deep learning phase correction and denoising with an anatomy conforming high-density-coil array can improve prostate diffusion imaging.

**Key Results** 

1. A high-density 50-channel pelvic coil substantially improves signal to noise and contrast to noise ratios compared with the standard 30-channel anterior array.
2. A prototype deep learning phase correction pipeline combined with a commercially available denoising reconstruction algorithm provides the greatest overall image-quality gains, particularly at high *b*-values.
3. Improved visualization of the prostate border, differentiation between peripheral and transition zones, lesion visibility, and confidence in identifying extraglandular tumor extension are possible.

## Introduction

Multiparametric MRI (mpMRI) is a cornerstone of prostate cancer screening and risk stratification.^1^ It is particularly effective in detecting clinically significant cancers, with reported sensitivity of 93% and specificity of 41%.^2^^,^^3^ Among mpMRI sequences, diffusion‑weighted imaging (DWI) provides the strongest association with clinically significant cancer and lesion conspicuity. High *b*-value DWI, combined with apparent diffusion coefficient (ADC), correlates more strongly with cancer grade and volume than T2-weighted (T2w) and dynamic contrast enhanced images.^4^^,^^5^ However, high *b*-value DWI is challenged by low signal-to-noise ratio (SNR) and susceptibility artifacts, such as wormholes from rectal gas, which obscure anatomy and hinder interpretation.^6^

Efforts to improve DWI quality span hardware and reconstruction. A conventional anterior array combined with a posterior array may not provide sufficient SNR because of the distance of coil elements from the prostate. Endorectal coils enhance image quality through proximity and stabilization, but are rarely used at 3 T because of discomfort, cost, and setup complexity.^7^ Recent advances in high-element pelvic arrays reduce coil-to-prostate distance and have shown improved SNR in phantom studies for T2w and DWI over conventional anterior array with posterior array.^8^ On the reconstruction side, signal averaging can increase SNR but at the expense of scan time and motion. Denoising techniques, particularly recent deep learning (DL) approaches, can also enhance SNR during reconstruction without extending scan time.^9^ Furthermore, a DL-based phase correction (DLPC) model was recently proposed to improve complex signal averaging, which is an integral step in DWI reconstruction.^10^^,^^11^

This study hypothesizes that combining recent advancements—a novel 50‑channel pelvic coil, a deep learning–based denoising algorithm, and a vendor investigational DL-based phase correction algorithm—will improve prostate DWI quality, particularly at higher *b*-value, whether acquired or synthesized. The aims are to evaluate the feasibility of integrating these developments and to determine the incremental benefits of each of the features compared with the current clinical configuration using objective metrics and reader-based assessments.

## Materials and methods

### Study population

This prospective institutional review board–approved (IRB21-1782), Health Insurance Portability and Accountability Act–compliant, single-center feasibility study enrolled and imaged 24 consecutive men referred for prostate mpMRI between December 1, 2023, and April 24, 2025. Patients with previous radiation or hormonal therapy were excluded from the study because of signal changes from treatment. All participants gave informed consent before imaging. Four subjects were excluded from analysis because of incomplete examinations or inadequate image quality. There was a loose coil element in 1 subject that led to poor image quality. One subject had an incomplete examination because of claustrophobia. One subject had severe bilateral hip arthroplasty artifacts, and 1 patient had a radical proctectomy.

### Coil details

**Figure 1:**
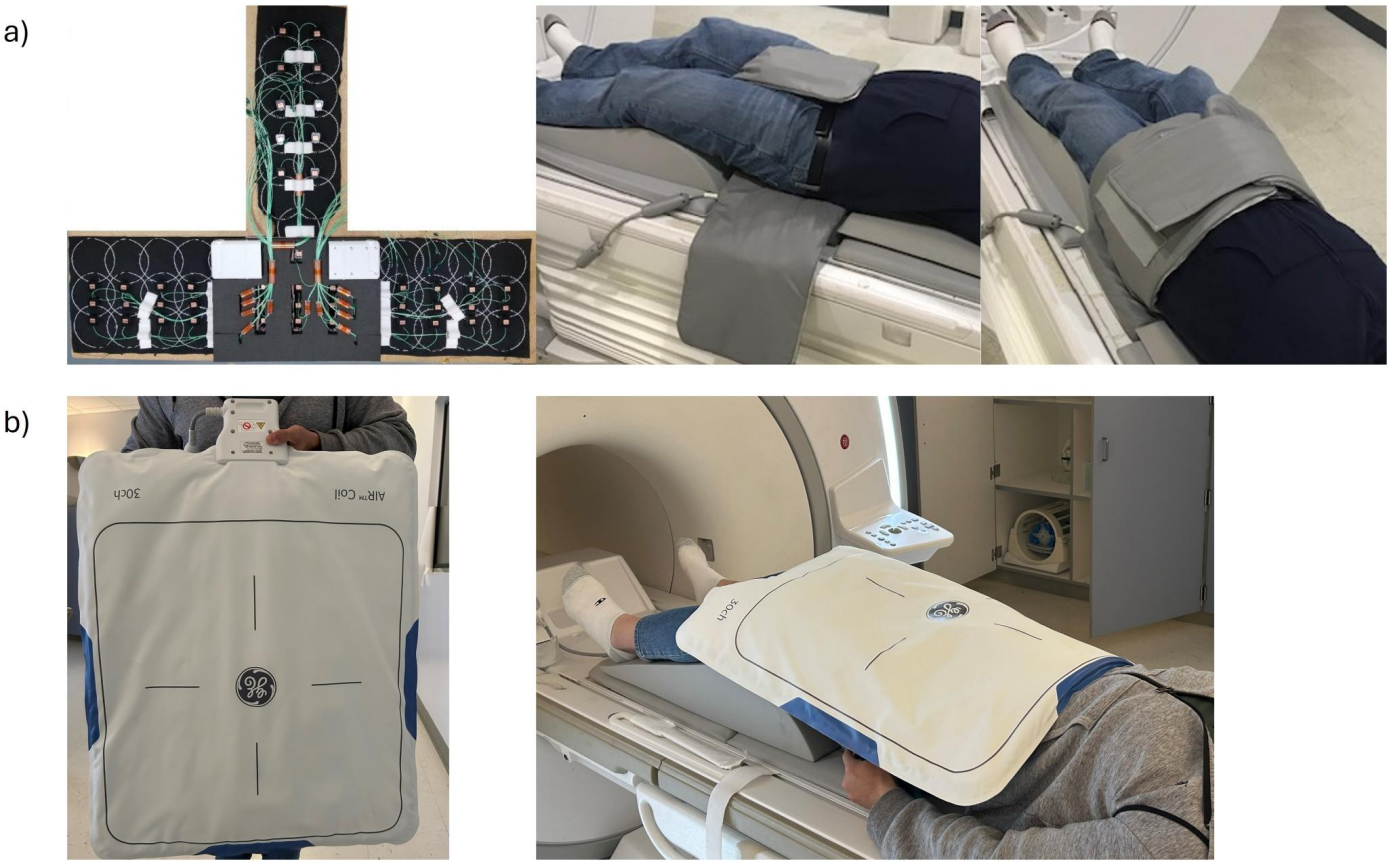
(a) The prototype coil element layout and patient configuration for the 50-channel pelvic coil (GE HealthCare, Waukesha, WI, USA) is illustrated. AIR coil loops are placed between 2 thermoplastic coated thin fabrics (0.6 mm). Dartex was used as a coil cover for biocompatibility and water seal. Full coverage of the prostate and rectal area was achieved by 50 elements located in a posterior base with 2 lateral flaps (left and right sides) and 1 center flap. The elements in the center flap ensure added coverage in the perineum, where standard anterior and posterior phase arrays cannot cover. (b) The commercially available 30-channel AIR coil anterior array and subject setup are illustrated.

The prototype pelvic coil (Figure 1a) was built using AIR coil loops^12^ on a flexible fabric. Loops were embedded between 2 thin (0.6-mm) thermoplastic-coated fabric sheets, enclosed in a biocompatible outer skin. Electronics modules—including preamplifiers, decoupling, matching, and baluns—were integrated with the loops. Nearest-neighboring elements were decoupled by overlapping and next-nearest-neighbor and more distant elements were decoupled by preamplifiers. The 50-channel (50ch) layout included a posterior base (9 channels), lateral flaps (15 channels each), and a center flap (11 channels) for full pelvis and perineal coverage.^8^

### Data acquisition

All MR examinations were obtained on a 3 T SIGNA Architect Scanner (GE HealthCare, USA). The 50ch pelvic coil (GE HealthCare, USA) was strapped around the patient’s pelvic region. Axial T2w fast spin echo images and DWI were acquired. Then a routine mpMRI was acquired using a standard 30-channel AIR Coil anterior array (AA) and posterior array (GE HealthCare, USA) (Figure 1b), including axial T2-weighted and DWI using scan parameters matching the 50ch acquisitions. T2w fast spin echo with 3-mm slice thickness, Echo Time of 180 ms, was acquired in 5 minutes. DWI scans were acquired using a reduced field of view, single-shot DWI sequence with 4 different *b*-values, a slice thickness of 3 mm, in 7:09 minutes. Full imaging parameters are listed in Table 1.

**Table 1:** Full imaging parameters.

	T2-weighted FSE	DWI
*b* value (s/mm^2^)	–	50 150 990 1500
TR (ms)	6800- 8000	6000–6700
TE (ms)	180	82
Slice thickness (mm)	3	3
Slice gap (mm)	3	3.3
Number of averages	1.5	2,2,7,10
Echo train length	26	–
Field of view (mm^2^)	200x200	214x200
Acquisition matrix	452x430	110x80
Scan time (min)	5	7:09
ARC	2	–

Abbreviations: ARC, autocalibrating reconstruction for Cartesian imaging acceleration factor; DWI, diffusion-weighted imaging; FSE, fast spin echo; TE, echo time; TR, repetition time.

### Reconstruction

The DWI acquisitions were reconstructed with a commercially available algorithm AIR Recon DL (ARDL)^13^ on the scanner with a denoising level “high.” The same raw data were also reconstructed offline using a prototype DLPC combined with ARDL (DLPC+ARDL), maintaining the same denoising level.

In standard product reconstruction, a low-pass filter is used to correct shot-to-shot phase variations before complex signal averaging. DLPC generates a high-quality phase (high resolution and SNR) for phase correction using a DL-based network, which is a U-Net residual network with 4.4 million trainable parameters in approximately 10 000 kernels. It was pretrained using supervised learning from a database of more than 10 000 images with various SNR levels and frequencies of background phases. DLPC was embedded in the product reconstruction pipeline by replacing the conventional low-pass filter-based phase correction before complex signal averaging. The model was distributed to the research community as part of a prototype diffusion reconstruction.^10^^,^^11^ Compared with a product low-pass filter-based phase correction, DLPC more effectively separates high-frequency background phase from phase noise, resulting in reduced signal bias. This has been demonstrated previously in digital phantoms, MRI phantoms, and multiple other clinical applications.^10^^,^^11^^,^^14–17^ Accurate phase correction also preserves the noise distribution, therefore reducing the noise floor and increasing SNR in the complex averaged images.^11^^,^^14^^,^^17–24^

All postprocessing—generation of ADC maps and synthetic *b *= 2000s/mm^2^ images per reconstructed set of DWI scans—was done on the reconstructed images using commercially available applications in READYView (GE HealthCare, USA) to analyze the impact of DL reconstruction and prototype coil on fitting algorithms via qualitative and quantitative image analysis described later.

Images acquired with the AA coil and reconstructed using the standard ARDL pipeline served as the clinical baseline. To isolate the impact of the high-density coil array, images from the same reconstruction pipeline were generated and compared for 2 coils under investigation, using the same or similar slice positions. To isolate the impact of DLPC reconstruction, images obtained with the 50ch coil, but reconstructed with the 2 available pipelines, were compared.

### Image analysis

#### Qualitative

All images were reviewed on a PACS workstation (Philips IntelliSpace Radiology, Netherlands) by 2 radiologists independently, 1 with 7 years of prostate MRI experience (G.L.) and the other with 3 years of prostate MRI experience (N.K.). Each reconstructed image set for each coil configuration, including the synthetic high *b-*value images, was evaluated for image quality on a per‑slice basis using a 5‑point scale (1 = worst, 5 = best). The readers scored DWI and ADC images together for: (1) prostate border visualization, (2) peripheral‑to‑transition zone distinction (PZ/TZ), (3) lesion conspicuity, and (4) confidence of extraprostatic extension (EPE). T2w images were used only as an anatomical reference and were not scored. The 4 combinations (AA with ARDL, AA with DLPC+ARDL, 50ch with ARDL, and 50ch with DLPC+ARDL) were reviewed independently without a washout period.

#### Quantitative image analysis

SNR and contrast-to-noise ratio (CNR) of the *b = *1500 s/mm^2^ image were estimated by placing an elliptical region-of-interest (ROI) on the suspicious lesion to obtain the mean signal value, and an ROI in the bladder to measure the SD of the signal as an estimate of image noise. One scientist with 9 years of MR experience drew the ROIs in consultation with the radiologist (G.L.) and performed the quantitative analysis. SNR was calculated as the mean lesion signal divided by the noise. CNR was calculated by the signal difference between the lesion and muscle, divided by image noise. SNR and CNR were not calculated for synthetic images, as the noise characteristics are not representative of true acquisition noise, making these quantitative metrics nonmeaningful for synthetic data.

### Statistical methods

The combined reader scores were compared using the Wilcoxon matched-pairs signed-rank test between combinations of AA with ARDL, AA with DLPC+ARDL, 50Ch with ARDL, and 50Ch with DLPC+ARDL. A *P* value of <.05 was considered statistically significant. Interreader variability was assessed by using quadratic-weighted Cohen’s κ.

All continuous measures are summarized as mean ± SD and were compared using the 2-sided paired Student *t*-test. Statistical analysis was performed in Matlab (MathWorks, USA), R version 4.5.2 (R Foundation for Statistical Computing, Austria), and Excel (Microsoft, USA).

### DLPL algorithm availability

Access to the prototype DLPC reconstruction pipeline can be requested by research collaborators for a limited-term evaluation at https://weconnect.gehealthcare.com/s/feed/0D53a00008uGMA7CAO

The tool is available to all users with a valid GE HealthCare (GEHC) system within their institution.

## Results

This study enrolled 24 men; however, 4 were excluded from analysis because of inadequate or incomplete imaging. The first subject produced highly noisy images, which were attributed to a disconnected coil element, which was immediately repaired before proceeding with additional scans. The coil element was replaced, and all joints were resoldered by the vendor to reinforce the coil connections. One subject had severe artifacts resulting from his bilateral hip arthroplasty. Another subject was unable to complete the examination because of claustrophobia and returned later for the clinical scan only. One subject had a prior prostatectomy. Two patients were excluded from quantitative image analysis because of mismatched acquisition parameters between 50ch and 30ch acquisitions. Table 2 summarizes the characteristics of the analyzed cohort along with additional details in supplementary figure 1.

**Table 2:** Cohort information of those included in the analysis.

Number of analyzable participants	20
Age (y)	68 ± 8
Prostate-specific antigen (ng/mL)	10.31 ± 12.56
BMI	29.09 ± 5.02
Number of days between MRI and biopsy	39.27 ± 17.40
Race	
Asian	0 (0)
Black	8 (40)
White	9 (45)
Unknown	2 (10)
Native American	1 (5)
Outcomes and cancer grade	
No biopsy	5
Benign	2
Gleason 3 + 3	4
Gleason 3 + 4	6
Gleason 4 + 3	0
Gleason 4 + 4	1
Gleason 4 + 5	2
TRUS fusion-guided core biopsy	13
Prostatectomy	2
No biopsy	5

Unless otherwise indicated, data are means ± SDs or numbers with percentages in parentheses.

Abbreviations: BMI, body mass index; TRUS, transrectal ultrasound.

**Figure 2:**
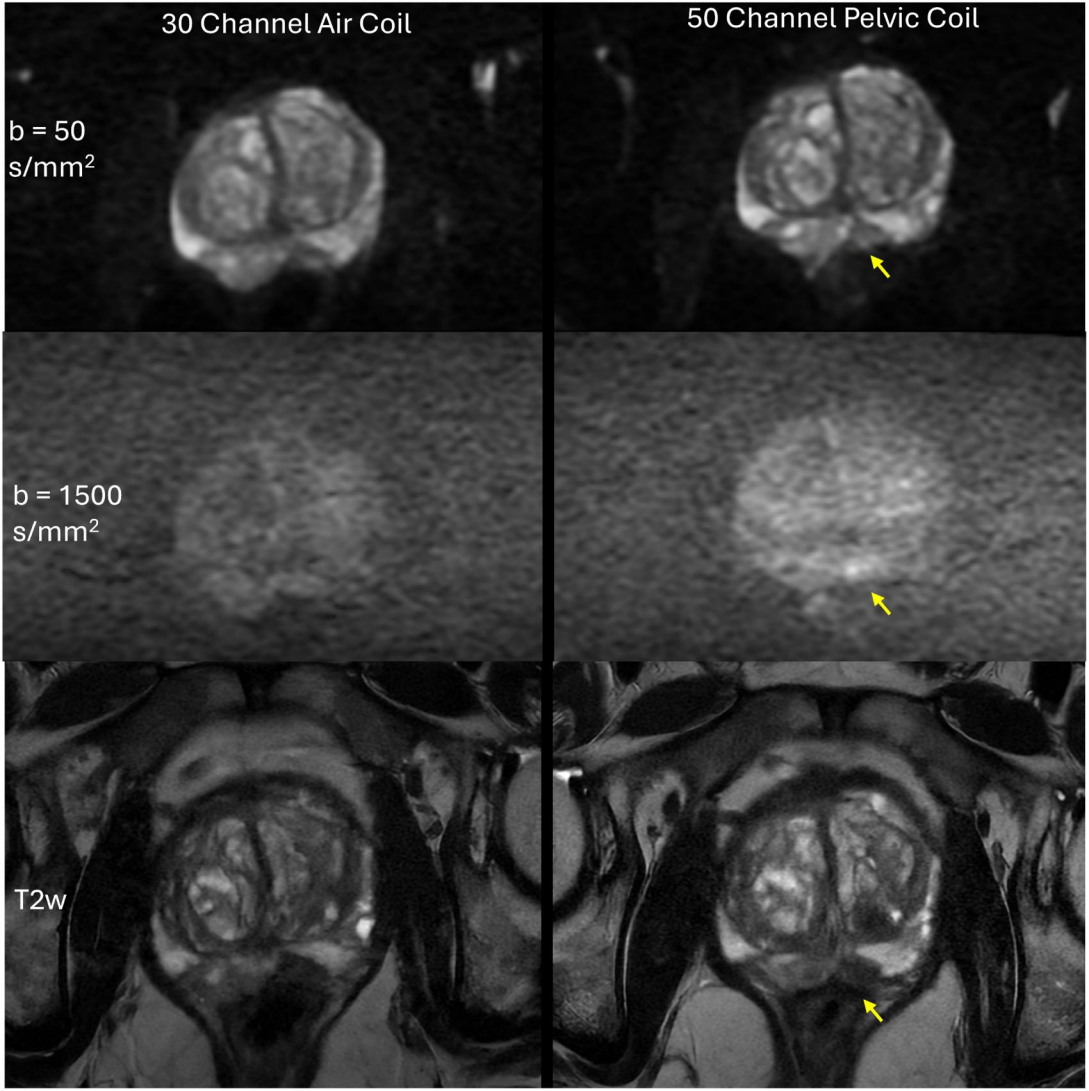
Diffusion-weighted images (DWI, *b *= 50 s/mm^2^, top; *b *= 1500 s/mm^2^, middle) and T2-weighted (T2w, bottom) images acquired using the product 30-channel AIR coil and posterior array (left) and the 50-channel pelvic coil (right) are shown. All images are reconstructed with product AIR Recon DL. This is a 71-year-old man, prostate-specific antigen = 4.72 ng/mL, with a left mid-gland posterior medial peripheral zone Gleason 3 + 3 lesion under active surveillance. Window and level are adjusted between the image pairs to ensure similar contrast despite different signal-to-noise ratio (SNR) because of coil differences. Overall, the 50-channel images have a higher perceived signal level. The *b = *50 s/mm^2^ images show better zonal distinction with the 50-channel coil. In the *b *= 1500 s/mm^2^ images, the suspected lesion (yellow arrow) has more hyperintense DWI signal with the 50-channel coil (middle, right), and the gland border is more distinct with the 50-channel pelvic coil than with the 30-channel AIR coil. Therefore, the 50-channel pelvic coil demonstrates modest enhancements in DWI. T2w images are used as an anatomical reference.

DWI scans acquired with the 50ch pelvic coil demonstrated a modest improvement in overall image quality compared with those obtained using the AA and reconstructed with the product ARDL pipeline. Although the 50ch provided a higher perceived signal, image noise remained substantial at high *b* value; the overall improvement in image quality remains limited. Figure 2 illustrates this observation.

**Figure 3:**
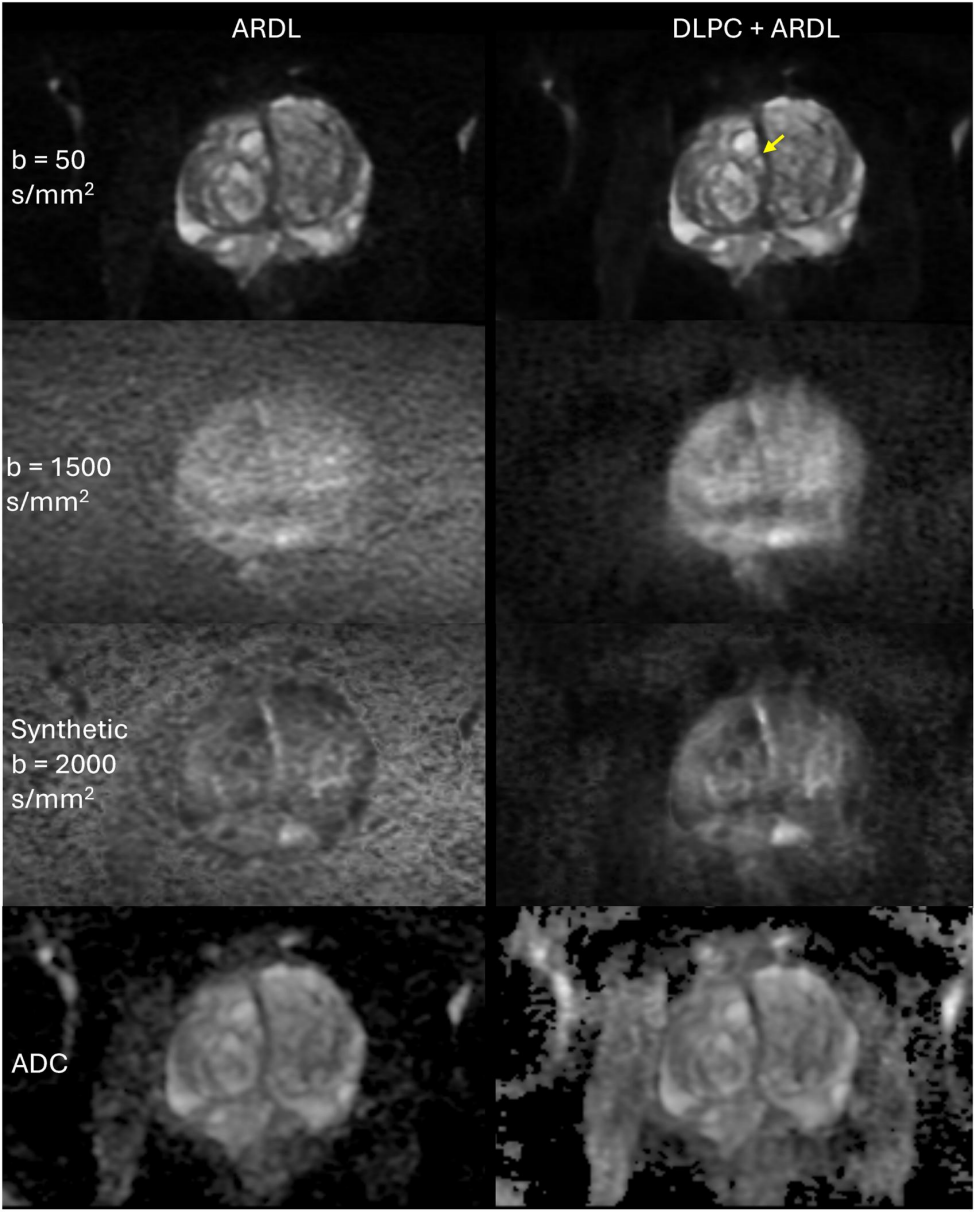
Diffusion-weighted images (DWI) acquired using the 50-channel pelvic coil (first and second row) reconstructed with product AIR Recon DL (ARDL) denoising (left) and DL Phase Correction with ARDL (DLPC + ARDL) (right), the corresponding synthetic *b = *2000 s/mm^2^ images (third row), and apparent diffusion coefficient (ADC) maps (bottom row) are shown with matched window and level between the image pairs. Compared to the improvements in Figure 2, the improvement resulting from DLPC reconstruction is much more significant. At *b = *50 s/mm^2^ (top row), small structural details show noticeable improvements in using DLPC+ARDL, such as a better delineation of the border of the benign nodules (yellow arrow). DLPC+ARDL reconstructed images have strong background noise suppression, particularly noticeable at *b = *1500 s/mm^2^ (second row), leading to a better delineation of the prostate gland and distinctive hyperintense lesion border. Improved image quality with DLPC reconstruction results in improved ADC estimation, evident in clear prostate gland delineation and clear border visualization in hypointense signal regions signifying restricted diffusion, and more homogenous signals in low SNR regions, such as muscles.

Applying DLPC demonstrates the ability to further reduce background noise using the same acquisition. Figure 3 shows the same subject reconstructed with both ARDL and DLPC+ARDL using the 50ch coil as the baseline. Although *b = *50 s/mm^2^ images show slight resolution improvement, *b = *1500 s/mm^2^ images reconstructed with DLPC+ARDL demonstrate a notable reduction in background noise, enhancing gland delineation, zonal distinction, and lesion conspicuity. The hyperintense lesion border also becomes more distinct.

**Figure 4:**
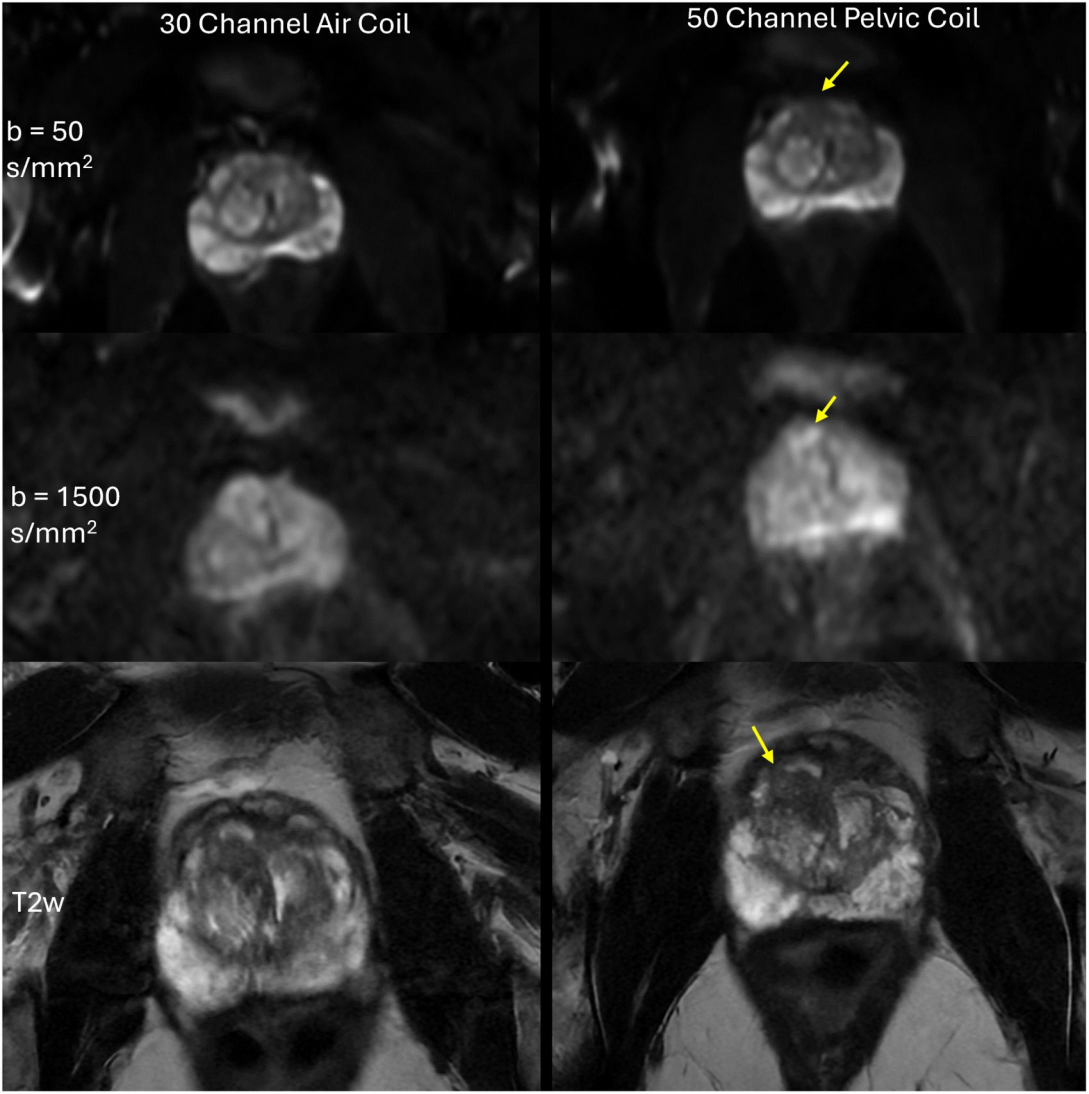
Diffusion-weighted images (DWI, top and middle) and T2-weighted (T2w, bottom) images acquired using the product 30-channel air coil and posterior array (left) and the 50-channel pelvic coil (right) are shown. All images are reconstructed with product AIR Recon DL. The patient is a 64-year-old man with a prostate-specific antigen = 5.02 ng/mL. A similar slice position is chosen. Right anterior apical transition zone Gleason 3 + 4 lesion (yellow arrow) is more well defined and hyperintense with the 50-channel coil at b = 1500s/mm^2^ (second row, right). Radiologist feedback suggests that this anterior lesion can be easily missed by urologists when using the images acquired by the 30-channel air coil.

Because the acquired DWI data are used to calculate ADC maps and synthetic high *b*-value images, enhancements in DWI scan quality are expected to propagate to the resulting fitting maps/images. As illustrated in Figure 3, the ADC maps generated using DLPC+ARDL exhibit a more uniform signal, particularly in the muscle, where diffusion is anatomically consistent, whereas it is uncharacteristically nonuniform in the ARDL reconstructed version. The synthetic *b = *2000 s/mm^2^ image reconstructed with ARDL exhibits significant background noise, resulting in poor gland delineation and limited contrast within the peripheral zone, whereas the image reconstructed with DLPC+ARDL shows increased conspicuity of restricted diffusion, or hyperintense DWI signal. In this case, the suspicious left mid-gland posterior medial peripheral zone lesion was pathologically confirmed as Gleason 3 + 3. Additional cases, including clinically significant cancers, are shown in Figure  4 and supplementary figure 2.

**Figure 5:**
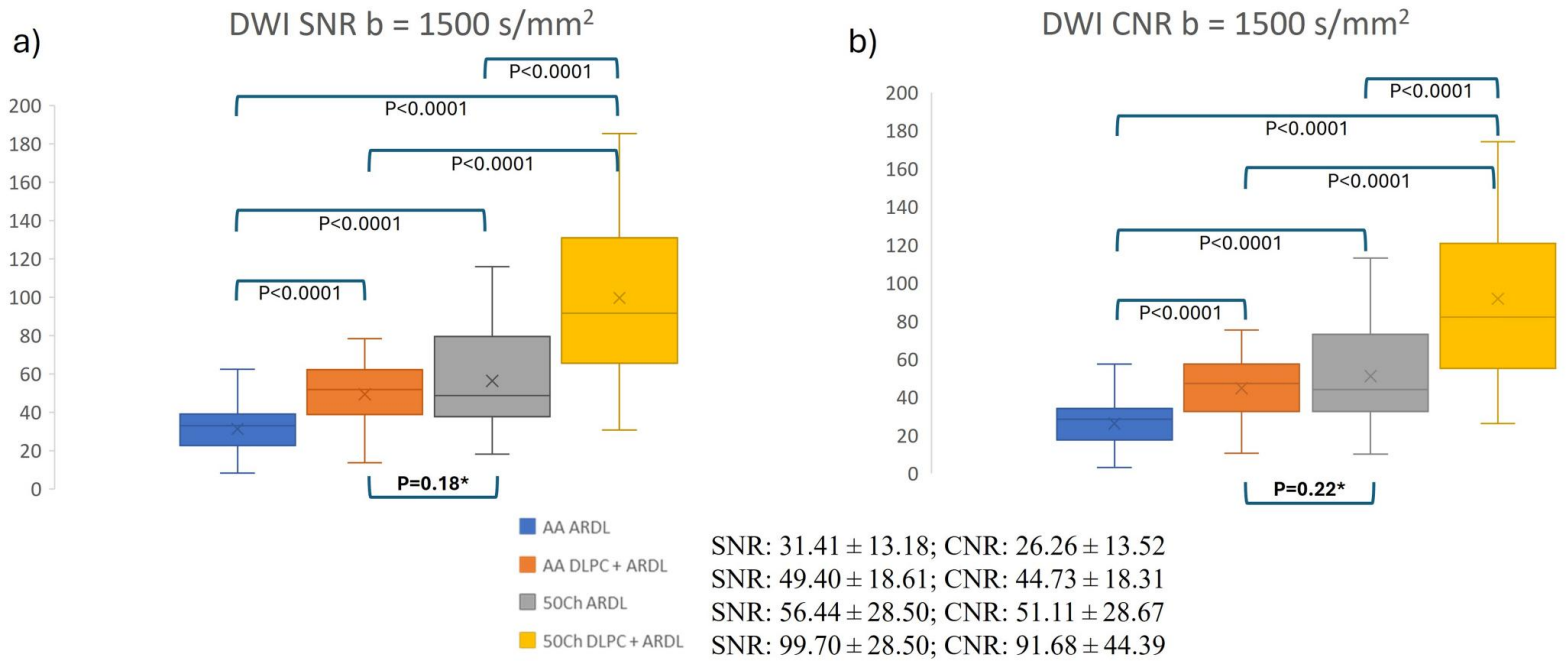
Boxplots of signal-to-noise ratio (SNR) (a) and contrast-to-noise ratio (CNR) (b) boxplots derived from diffusion-weighted images (*b *= 1500 s/mm^2^) acquired using either a 30‑channel anterior array (AA) or a 50‑channel pelvic coil (50ch). Each dataset was reconstructed with AIR Recon DL denoising (ARDL) or with deep learning phase correction combined with ARDL (DLPC + ARDL). SNR and CNR mean ± SD values for each category are reported within the figure key, and statistical comparisons between categories are indicated to highlight significant differences (or the lack of) between each configuration.

**Figure 6:**
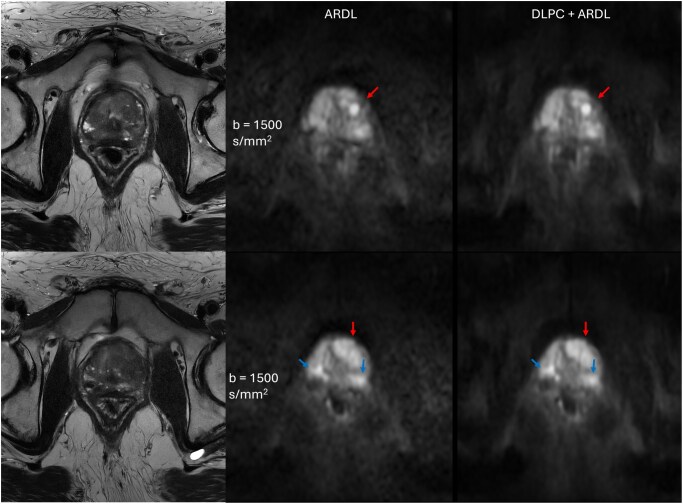
Patient images demonstrating improved small detail visibility in diffusion weighted images (DWI) reconstructed with DL Phase Correction with ARDL (DLPC + ARDL, right) in comparison to product AIR Recon DL (ARDL, middle) for detection of multifocal lesions and extraprostatic extension in the base (top) and midgland (bottom). Anatomical references are shown in the T2-weighted (T2w) images (left). This patient is 61 years old with a prostate-specific antigen  = 14.8 ng/mL, with a large transition zone cancer (Gleason 4 + 3) that almost extends to the capsule (red arrow) and was pathologically confirmed with the presence of extraprostatic extension along with left and right peripheral zone cancers (blue arrows). The left peripheral zone Gleason 4 + 3 cancer touching the left neurovascular bundle had a pathology-confirmed presence of perineural invasion. With DLPC + ARDL reconstruction, the reduced noise levels improve visibility of smaller structural details within the right peripheral zone (Gleason 4 + 5) lesion.

Quantitative metrics of SNR (Figure 5a) and CNR (Figure 5b) further support these findings. SNR from the 50ch coil with ARDL was statistically significantly higher than that of AA with ARDL (56.44 ± 28.50 vs 31.41 ± 13.18, *P *< .0001). SNR from the 50ch coil with DLPC+ARDL shows a statistically significant improvement over SNR from the 50ch coil with ARDL alone (99.70 ± 28.50 vs 56.44 ± 28.50, *P *< .0001). CNR values demonstrated a comparable pattern, with the 50ch DLPC+ARDL configuration outperforming all other combinations. As an example, Figure 6 highlights the superior anatomic details achieved with DLPC+ARDL reconstruction applied to data acquired using the 50ch pelvic coil. DLPC can also have an impact on motion-induced wormhole artifacts, which are highlighted in supplementary figure 3.

Combined scores from the 2 radiologists showed significant, stepwise improvements in both *b *= 1500 s/mm^2^ and ADC image quality across all metrics when moving from the AA coil with ARDL to the 50ch coil with DLPC+ARDL. For DWI quality scores, compared with AA with ARDL, the 50ch with DLPC+ARDL configuration yielded higher scores for all scoring categories (all *P *< .0001). 50ch with DLPC+ARDL also significantly improved DWI scores relative to 50ch with ARDL alone (*P *= .002–.02). For ADC quality, a similar pattern was observed. 50ch with DLPC + ARDL significantly outperformed AA with ARDL (all *P *< .0001). 50ch DLPC+ARDL provided additional gains over 50ch ARDL for prostate border visualization, peripheral zone/transition zone (PZ/TZ) distinction, and confidence of EPE (*P *= .03–.04); however, there was no statistically significant improvement in lesion conspicuity (*P *= .25). Similarly, combined scores demonstrated significant improvements in synthetic DWI (*b *= 2000 s/mm^2^) image quality across all metrics when transitioning from AA with ARDL to 50ch with DLPC+ARDL. Compared with AA with ARDL, 50ch with DLPC+ARDL configuration yielded higher average scores (all *P *< .0001). Within the 50ch coil configuration, DLPC+ARDL further improved averaged synthetic DWI scores over 50ch ARDL and reached statistical significance for prostate border visualization, PZ/TZ distinction, and confidence of EPE (*P *= 6.1 × 10^−5^–0.01), whereas the difference for lesion conspicuity did not reach significance (*P *= .06). A full summary is provided in Tables 3 and 4. Overall, the results demonstrate synergistic benefits from the high‑density 50ch coil and DLPC‑based reconstruction, with the 50ch DLPC+ARDL combination achieving the highest image quality scores across all metrics.

**Table 3:** Reader scores for image quality (using a 5‑point scale: 1 = worst, 5 = best) for *b = *1500 s/mm^2^ diffusion-weighted imaging series.

		AA ARDL^1^	AA DLPC^2^	50 CH ARDL ^3^	50 CH DLPC^4^	*P* value			
						AA ARDL vs AA DLPC	AA ARDL vs 50 CH ARDL	AA ARDL vs 50 CH DLPC	50 CH ARDL vs 50 CH DLPC
ADC quality									
	Prostate border visualization	3.72 ± 0.88	3.97 ± 0.83	4.33 ± 0.80	4.49 ± 0.67	**.002**	**2.5e-5**	**7.1e-6**	**.04**
	PZ/TZ distinction	3.56 ± 0.87	3.79 ± 0.88	4.33 ± 0.83	4.49 ± 0.75	**.004**	**8.9e-6**	**1.4e-6**	**.04**
	Lesion conspicuity	3.62 ± 0.87	3.79 ± 0.76	4.44 ± 0.74	4.51 ± 0.67	**.02**	**8.9e-5**	**2.9e-5**	.25
	Confidence of EPE	3.59 ± 0.90	3.74 ± 0.93	4.41 ± 0.71	4.54 ± 0.67	*.07*	**1.7e-5**	**4.2e-6**	**.03**
DWI (*b* = 1500 s/mm^2^) quality									
	Prostate border visualization	3.33 ± 0.86	3.64 ± 0.83	4.00 ± 0.91	4.26 ± 0.90	**9.8e-4**	**4.5e-6**	**2.0e-6**	**.002**
	PZ/TZ distinction	3.23 ± 0.15	3.51 ± 0.96	3.87 ± 0.94	4.10 ± 0.90	**9.8e-4**	**2.6e-5**	**4.2e-6**	**.004**
	Lesion conspicuity	3.26 ± 1.13	3.69 ± 0.99	4.03 ± 0.89	4.38 ± 0.77	**9.6e-5**	**3.1e-5**	**2.6e-6**	**2.4e-4**
	Confidence of EPE	3.08 ± 1.02	3.44 ± 1.01	4.00 ± 0.96	4.18 ± 0.93	**2.4e-4**	**1.2e-5**	**2.6e-6**	**.02**

Bolded values indicate statistical significance (*P *< .05) with comparison as labeled by the superscript in the title. Italicized values indicate close to statistical significance.

Abbreviations: AA ARDL¹, 30‑channel anterior array with AIR ReconDL denoising; AA DLPC², 30‑channel anterior array with deep‑learning phase correction plus AIR ReconDL; 50 CH ARDL³, 50‑channel pelvic coil with AIR ReconDL; 50 CH DLPC⁴, 50‑channel pelvic coil with deep‑learning phase correction plus AIR ReconDL; EPE, extraprostatic extension; PZ/TZ, peripheral zone/transition zone.

**Table 4:** Reader score for image quality (using a 5‑point scale: 1 = worst, 5 = best) for *b = *2000 s/mm^2^ diffusion-weighted imaging series.

		AA ARDL^1^	AA DLPC ^2^	50 CH ARDL^3^	50 CH DLPC^4^	*P* value			
						AA ARDL vs AA DLPC	AA ARDL vs 50 CH ARDL	AA ARDL vs 50 CH DLPC	50 CH ARDL vs 50 CH DLPC
Synthetic DWI (*b* = 2000s/mm^2^) quality									
	Prostate border visualization	3.00 ± 0.82	3.62 ± 0.77	3.62 ± 0.87	4.03 ± 0.80	**3.6e-6**	**3.4e-6**	**7.5e-7**	**6.1e-5**
	PZ/TZ distinction	2.90 ± 0.90	3.36 ± 0.89	3.49 ± 0.81	3.82 ± 0.84	**1.7e-4**	**7.3e-5**	**2.6e-6**	**2.4e-4**
	Lesion conspicuity	3.51 ± 1.01	3.85 ± 0.95	4.03 ± 0.95	4.21 ± 0.79	**9.8e-4**	**2.1e-4**	**5.8e-5**	*.06*
	Confidence of EPE	3.33 ± 1.07	3.69 ± 0.91	3.87 ± 0.97	4.08 ± 0.83	**4.9e-4**	**1.8e-4**	**1.6e-5**	**.01**

Bolded values indicate statistical significance (*P *< .05) with comparison as labeled by the superscript. Italicized values indicate close to statistical significance.

Abbreviations: AA ARDL¹, 30‑channel anterior array with AIR ReconDL denoising; AA DLPC², 30‑channel anterior array with deep‑learning phase correction plus AIR ReconDL; 50 CH ARDL³, 50‑channel pelvic coil with AIR ReconDL; 50 CH DLPC⁴, 50‑channel pelvic coil with deep‑learning phase correction plus AIR ReconDL; EPE, extraprostatic extension; PZ/TZ, peripheral zone/transition zone.

Inter‑reader agreement was assessed for both the acquired high‑*b*‑value DWI (*b *= 1500 s/mm^2^) and synthetic DWI (*b *= 2000 s/mm^2^) series. For the *b* = 1500 s/mm^2^ scoring pairs, the 2 readers demonstrated fair agreement, with a quadratic‑weighted κ of 0.34 (95% CI, 0.28–0.40). Agreement for the synthetic *b* = 2000 s/mm^2^ images was lower, with a quadratic‑weighted κ of 0.19 (95% CI: 0.10–0.27).

## Discussion

In summary, this study demonstrates that combining the 50ch pelvic coil with deep learning–based denoising and phase correction meaningfully, qualitatively and quantitatively improves prostate DWI scan quality. The 50ch coil consistently outperformed the standard anterior array across all qualitative metrics from radiologists’ scores. The increased signal level from the high-element coil, together with the deep learning phase-correction component, further reduced background noise and enhanced image quality, particularly at high *b* values. Collectively, these findings show that advances in both hardware and reconstruction provide synergetic benefits, with the combination of a high‑density coil and DLPC + ARDL reconstruction offering the highest overall prostate DWI quality.

Beyond improvements in the acquired DWI series, this study also shows that higher input image quality from the 50ch coil and DLPC+ARDL reconstruction leads to more robust ADC map estimation, especially in low SNR regions. These gains propagated to synthetic high *b*‑value images, which demonstrate greater border, zonal, and lesion distinction and lesion conspicuity. Synthetic DWI is accepted as a substitute for acquired high *b*-value DWI to decrease image acquisition time and high *b*-value signal loss. This method could improve confidence in synthetic *b* images by optimizing input image quality. Hence, another benefit would be to enable high-quality synthetic *b* images without the need to acquire ultrahigh *b*-value (≥1400 s/mm^2^) images, reducing overall acquisition time. This technique has the potential to improve prostate diffusion imaging at lower field strengths (eg, 1.5 T) where the SNR is inherently lower than 3 T, especially without endorectal coils. While further quantitative analysis and histopathology comparison are needed to draw conclusions on diagnostic performance, an improvement can be plausibly expected.

Interreader agreement ranged from fair to low, reflecting the known subjectivity of qualitative image assessment and potential differences in reader preference. One reader consistently gave higher scores than the other reader, reflecting personal preference, even though scoring criteria were predefined. Nonetheless, both readers exhibited consistent directional trends, indicating that although absolute scores differed, the relative benefits of the 50ch coil with DLPC + ARDL remain robust across readers.

Previous work to improve prostate DWI has included enhancing signal through advanced coil design and improving reconstruction performance. Recent advancements in the 50ch pelvic coil reduce coil-to-prostate distance and have shown improved SNR in phantom studies for T2w and DWI over conventional anterior array with posterior array.^8^ In vivo results from this study mirror those findings.

On the reconstruction side, deep learning denoising methods similar to ARDL have been shown to improve prostate DWI quality.^25^ Most generalized DL denoising methods operate on complex-valued images to optimize noise removal in high-noise scenarios. Shot-to-shot phase variations in DWI prohibit direct complex signal averaging because this causes unwanted signal cancellation. Phase correction can remove incoherent phase variations and enable complex signal averaging. However, the parameters of the conventional phase correction (filter-based and adaptive) methods need to be adapted to the acquisition and reconstruction parameters for optimal phase correction.^26–28^ Suboptimal phase correction can lead to artifacts, biased signals, and complex noise distributions, undermining the performance of denoising methods. Deep learning–based phase error correction has been proposed to estimate the background phase to improve the velocity estimation in 4-dimensional MRI.^29^ However, this model was combined with polynomial regression and is limited to estimating the low-frequency background phase. Furthermore, it is only compatible with abdominopelvic 4-dimensional-MRI. The DLPC model used in this work is a generalized phase correction model, which directly generates a high-resolution phase map and is compatible with all anatomies. It has shown robust phase correction at various noise levels and frequencies of phases, while preserving Gaussian noise distributions, reducing noise floor and enhancing the performance of DL denoising in DW imaging.

This study has several limitations. Diagnostic accuracy was not directly assessed, as a substantial portion of the cohort lacked histopathologic confirmation; 5 of the 20 analyzed patients did not undergo biopsy, and only 2 proceeded to prostatectomy, limiting the ability to correlate imaging findings with ground‑truth pathology. Second, because the study was designed to evaluate the impact of a prototype coil and a prototype DLPC+ARDL reconstruction pipeline on image quality, the influence of varying acceleration factors was not examined. Although the increased channel count should theoretically support higher acceleration without compromising image quality, a dedicated study is needed to assess its performance under highly accelerated conditions and to determine the associated workflow and economic implications, including potential reduced scan time and improved clinical throughput. Finally, the prototype coil used in this study is not yet of product quality. Of the 4 excluded datasets, 1 was attributed to a coil malfunction at study initiation, likely caused by shipping-related handling. Such issues are more common in prototype hardware; however, they are unlikely in clinical practice, where production coils must meet stringent regulatory and manufacturing standards. The remaining exclusions were due to patient-related factors and were unrelated to the coil design. Additional product-quality units will be required before initiating larger or multicenter studies to fully establish the clinical significance, reliability, and generalizability of the technology.

In conclusion, acquisition of DWI images using the novel prototype 50ch pelvic coil and reconstructed with deep learning–based denoising and phase correction pipeline yields the highest overall image quality metrics, including the current standard of care 30ch pelvic coil imaging. Combining SNR recovered from coil proximity to anatomical regions of interest and reduced noise from reconstruction, this technique demonstrates improved image quality of 1 of the most important sequences in mpMRI in the prostate.

## Supplementary Material

umag019_Supplementary_Data

## Data Availability

Data were acquired at University of Chicago Medicine, 5841 S. Maryland Ave, Chicago, IL 60637. Data generated or analyzed during the study are available from the corresponding author by request; the data can be shared under a valid data sharing agreement due to legal and ethical constraints.
