# Supplementary material for: Improved prostate diffusion imaging using deep learning denoising and phase correction with ultra-high-density coil array
Source: Radiol Adv. 2026 Mar 28;3(2):umag019. doi: 10.1093/radadv/umag019 (PMC13131225; doi:10.1093/radadv/umag019)
Supplement: umag019_Supplementary_Data [file umag019_supplementary_data.zip › Supplementary Material.pdf]

Supplementary Materials Title Page

Manuscript Title:

Improved Prostate Diffusion Imaging Using Deep Learning Denoising and Phase Correction With Ultra-High-Density Coil Array

Authors:

1. Sherry Huang, S, PhD, Research and Scientific Affairs, GE HealthCare, Rochester, MN, USA
2. Xinzeng Wang, PhD, MR Clinical Solutions & Research Collaborations, GE HealthCare, Houston, TX, USA
3. Patricia Lan, PhD, GE HealthCare, Menlo Park, CA, USA
4. Milica Medved, PhD, Department of Radiology, University of Chicago, Chicago, IL, USA
5. Nurullah Kaya, MD, Department of Radiology, University of Chicago, Chicago, IL, USA
6. Clyve Follante, K, BS, MR Coils, GE HealthCare, Aurora, OH, USA
7. Yunjeong Stickle, PhD, MR Coils, GE HealthCare Coils, Aurora, OH, USA
8. Jonathan Taylor, RT(R)(MR), Department of Radiology, University of Chicago, Chicago, IL, USA
9. Ambereen Yousuf, MBBS, Department of Radiology, University of Chicago, Chicago, IL, USA
10. Roger Engelmann, MS, Department of Radiology, University of Chicago, Chicago, IL, USA
11. Fraser Robb, J.L, PhD, MR Clinical Solutions & Research Collaborations, GE HealthCare, Aurora, OH, USA
12. Arnaud Guidon, PhD, MR Clinical Solutions & Research Collaborations, GE HealthCare, Boston, MA, USA
13. Grace Lee, MD, Department of Radiology, University of Chicago, Chicago, IL, USA
14. Aytekin Oto, MD, MBA, Department of Radiology, University of Chicago, Chicago, IL, USA

Corresponding Author:

Grace Lee, glee@bsd.uchicago.edu, (773) 702-6478, 5841 S. Maryland Avenue MC 2026, Chicago, IL, 60637

Table of Contents

- Supplementary Figures 1-3
- Supplementary Methods

Supplementary Figures

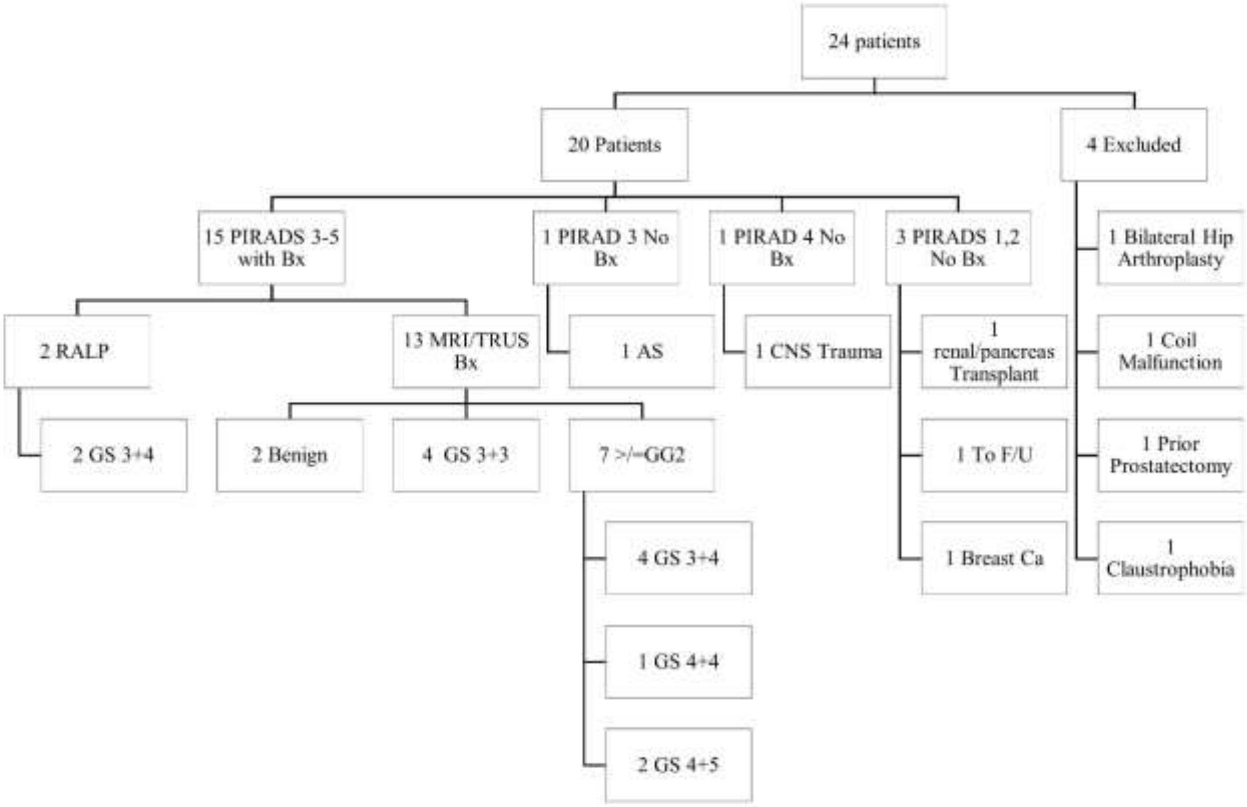

Figure S1. illustrating the composition of the patient cohort and subsequent clinical progression. The diagram details inclusion and exclusion criteria, imaging findings, biopsy (Bx) results, and downstream management pathways, including active surveillance (AS), as well as additional confounding clinical factors. One patient is pending organ transplantation, one patient is awaiting breast cancer (Breast Ca) treatment, and one patient has central nervous system (CNS) trauma and is awaiting follow-up (F/U) prostate-specific antigen (PSA) testing to determine whether biopsy is indicated. Two patients underwent robotic-assisted laparoscopic prostatectomy (RALP), and thirteen patients underwent MRI-guided or transrectal ultrasound (TRUS)-guided biopsy. For patients who underwent RALP or biopsy, Gleason score (GS) and Grade Group (GG) information is reported in the figure.

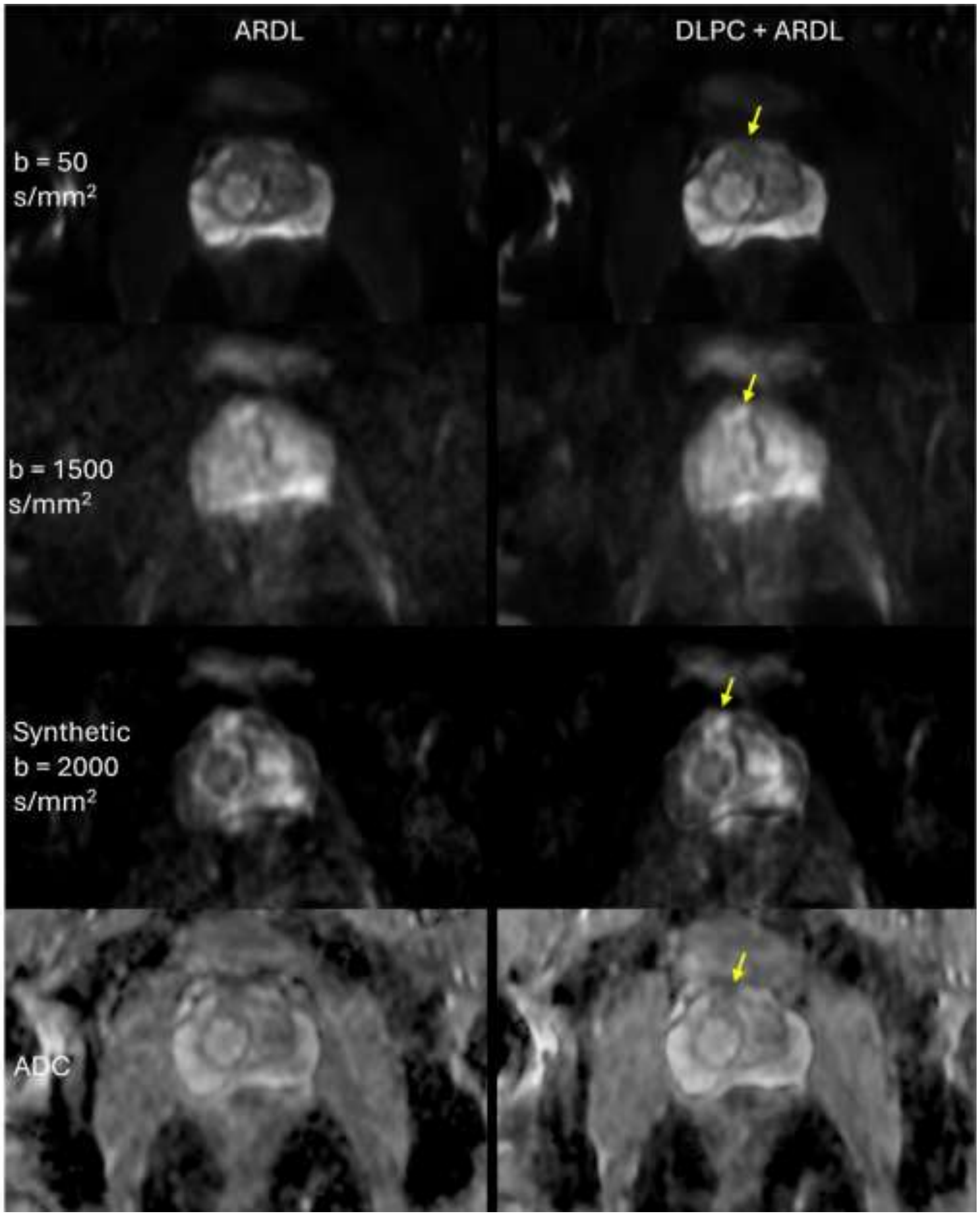

Figure S2. Diffusion-weighted images (DWI) acquired using the 50-Channel pelvic coil reconstructed with product AIR Recon DL (ARDL) denoising (left) and DL Phase Correction with ARDL (DLPC + ARDL) (right). The corresponding synthetic b=2000 s/mm^2 images, and ADC maps are shown with matched window and level between the image pairs. At b=1500 s/mm^2, there is noticeable improvement in lesion conspicuity (yellow arrow). DLPC + ARDL reconstructed images have better background noise suppression. Improved image quality with DLPC reconstruction results in better synthetic b=2000 s/mm^2 images, evident in clear border visualization in hyperintense signal region signifying restricted diffusion. This is because of improved ADC estimation in low SNR regions, such as muscles with short T2. This subject is smaller than the patient shown in figures 2-3, therefore the background noise overall was less.

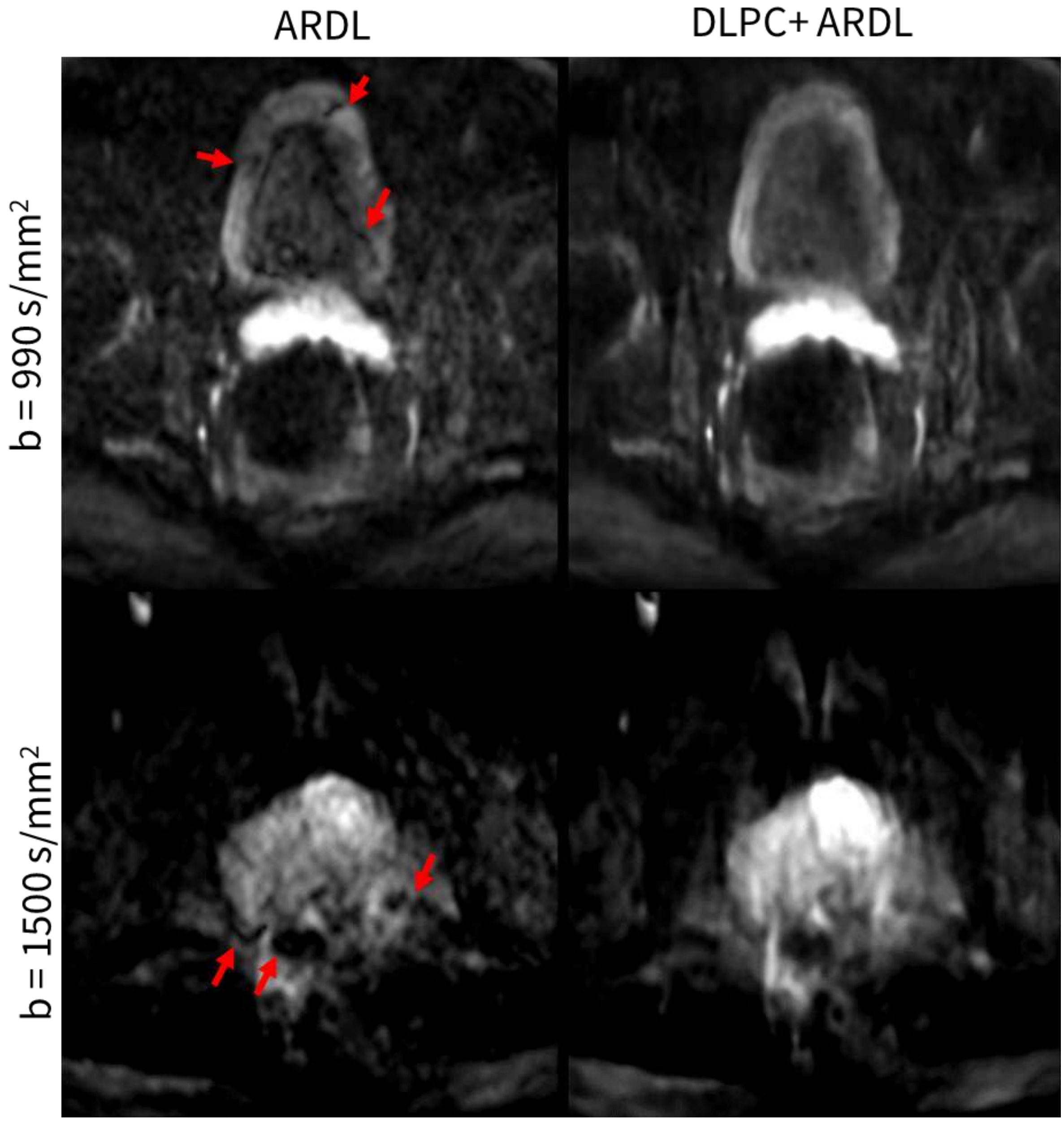

Figure S3: Diffusion-weighted imaging (DWI), particularly at high *b*-values, is susceptible to signal cancellation—often referred to as the wormhole artifact—caused by rapid phase variation due to motion. Red arrows indicate instances of the wormhole artifact in the bladder wall, rectal wall, and prostate area. AIR™ Recon DL reconstructed images still suffer from this behavior, whereas DL Phase Correction with ARDL (DLPC + ARDL) overcomes this issue. The bladder wall and prostate signal have been recovered, effectively eliminating the wormhole artifact.

Supplementary Methods:

All DICOM and raw data were anonymized in accordance with HIPAA safe-harbor guidelines. DICOMs were anonymized by the institution's Human Imaging Research Office (HIRO), while raw data were anonymized using a vendor-provided custom tool approved by HIRO.

Access to the prototype DLPC reconstruction pipeline can be requested by research collaborators for limited-term evaluation at
https://weconnect.gehealthcare.com/s/feed/0D53a00008uGMA7CAO
The tool is available to all users with a valid GEHC system within their institution.
